# The Ontario Cancer Research Ethics Board: a central REB that works

**DOI:** 10.3747/co.2008.196

**Published:** 2008-01

**Authors:** M.R. Chaddah

**Keywords:** Clinical trials, multicentre cancer protocols, research ethics board, reb, board of record, facilitated review, serious adverse events, sae

## Abstract

The Ontario Cancer Research Ethics Board (ocreb) has made its mark within Ontario as a successful, centralized, oncology-specific research ethics board. As such, ocreb has proven invaluable to principal investigators, sponsors, and study participants given its ability to reduce duplication during the submission process, to provide the highest quality of review, to shorten study start-up time, and to implement more efficient methods of reporting serious adverse events.

## 1. INTRODUCTION

The seed from which the Ontario Cancer Research Ethics Board (ocreb) sprang was planted in 2001. At the time, Raphael Saginur, an Ottawa-based clinical scientist and research ethics board (reb) chair, together with the Ontario Cancer Research Network, recognized the growing need to improve and streamline scientific and ethical reviews of multicentre cancer trials. They were not alone in their assessment; many other groups around the world were also experimenting with coordinated reb reviews, and precedents for centralized rebs did exist, meeting with mixed success 1,a.

Nurturing the idea took two years of thorough consultations among reb chairs, cancer centres, hospitals, researchers, bioethicists, clinical research coordinators, sponsors, and many collaborating centres (Table I). Those dialogues identified key issues that still apply today and that buttress support for the creation of a centralized, oncology-specific reb.

The major challenges faced by all ethics boards are the growing volume of clinical trials and the increasingly complex regulations governing rebs. Coping with the resulting increase in workload to review, approve, and monitor clinical trials is a significant burden. The rebs must find and retain appropriately qualified members, secure adequate resources and office staff, and respond to investigators in a timely fashion. Ensuring consistency of reviews across rebs is also an issue for multicentre studies. Such firmly rooted needs propelled the decision to move ocreb forward.

The organization’s framework took shape very quickly, with the chair, vice-chair, and fourteen reb members being recruited within four to five months. Under the aegis of a provincial program promoting clinical research, ocreb was officially launched in December 2003. At that time, ocreb’s funding flowed from the Ontario Cancer Research Network, whose programs were subsequently incorporated into the Ontario Institute for Cancer Research (oicr) in 2005. Currently, ocreb is funded at arm’s length by oicr and is independent of both the researchers conducting the clinical trials and the organizations sponsoring the research. It is accountable to an Advisory Committee that liaises with oicr’s board of directors.

## 2. ROLE OF OCREB

To appreciate the unique position that ocreb occupies in cancer clinical trials in Ontario, a review of the sponsor –investigator–reb relationship is worthwhile.

Sponsors typically develop and fund a study, coordinate the regulatory submissions (for example, to Health Canada), select the sites (often globally), monitor study progress, and manage the data analysis and study reporting. Many studies are sponsored by pharmaceutical or biotechnology companies that develop the therapies being evaluated. Cooperative groups such as the Clinical Trials Group of the National Cancer Institute of Canada also sponsor studies and run them through multiple sites established across the country. As well, the Princess Margaret Hospital (pmh) Phase ii Consortium sponsors studies that are conducted at four major cancer centres in Ontario, including pmh, and at centres in British Columbia, Alberta, and the United States. Investigators may also serve as sponsors.

Regardless of sponsorship, every site participating in a multicentre trial must gain approval from a reb. That process is set in motion by the principal investigator (pi) at each site. The pi submits the study protocol and supporting documents to the local institutional reb. The reb reviews and may request clarifications or changes to the protocol and consent form before granting approval to run the trial.

The site pi is also responsible for the conduct of the trial at the site, including follow-up and reporting of all medical incidents, termed adverse events (aes), to the sponsor and the reb. Serious aes (saes) warrant special scrutiny and must be reported in timely fashion. The reb is accountable to the institution for initial and ongoing ethical review and oversight of the research, a process that may result in disapproval, modifications to approved research, temporary shutdowns, or termination of the research project if deemed necessary.

As with any reb, ocreb is charged with the task of protecting volunteers who participate in clinical trials by ensuring respect for human dignity, free and informed consent, privacy and confidentiality, justice and inclusiveness, and balance in the harms and benefits of treatments. The qualities that distinguish ocreb from existing rebs stem from a versatile combination of its centralization, specialization, and management.

Centralization allows ocreb to serve as the reb for multiple institutions and reduces duplication during the submission process. For example, in a multicentre trial in which three sites retain ocreb, only one site investigator, deemed the “provincial applicant,” is required to embark on the full submission process on behalf of the province. Upon ocreb granting provincial approval, the sites are either approved in parallel if their abbreviated centre-specific applications are received at the same time as the provincial application, or are given expedited approval if they submit at a later date. Additional centres can easily join the study at any point during the process, and all participating centres agree to use the same consent form unless institutional policy requires specific local changes. Thus ocreb lessens the workload for the submitters and not only expedites but also coordinates the study start-up at multiple sites across the province. Centralization also permits safety issues to be addressed simultaneously, given that safety updates and amendments to the protocol and consent form apply equally at each site.

The benefits of centralization synergize with ocreb’s specialization in oncology. The board comprises seventeen members with a broad range of expertise in oncology, clinical research, biostatistics, epidemiology, pharmacology, research ethics, law, privacy, and community issues. This group offers applicants the highest quality of review and oversight. With greater knowledge and expertise in cancer than institutions with more generalized rebs, ocreb can provide a better context in which to oversee trials in specialized areas of oncology.

Underpinning the foregoing distinguishing qualities is ocreb’s strategic management, developed under the expert leadership of the executive director, Janet Manzo, and with guidance from the chair of ocreb, Dr. Ron Heslegrave, and the chair of ocreb’s Advisory Committee, Dr. Raphael Saginur. Together with ocreb’s staff, these individuals have worked to preserve a level of internal flexibility that has allowed the organization to refine its operational strategies as it gained experience. Paramount to ocreb’s success has been its ability to foster collaboration within the research ethics community through communication, exchange of information, and promotion of change.

In theory, the combination of all its assets should place ocreb in the sought-after position of being able to streamline the work of pis, participating centres, and institutional rebs. In practice, ocreb is living up to that potential. In ocreb’s first year of operation, institutions retained it either for a “facilitated review” or as their reb “of record.” For the former option, ocreb provides an expert review of the study to assist the local reb, which remains responsible for the study within the institution. For the latter option, ocreb enters into a contract with the institution to act as its reb on a study-by-study basis for initial and ongoing review.

**FIGURE 1:**
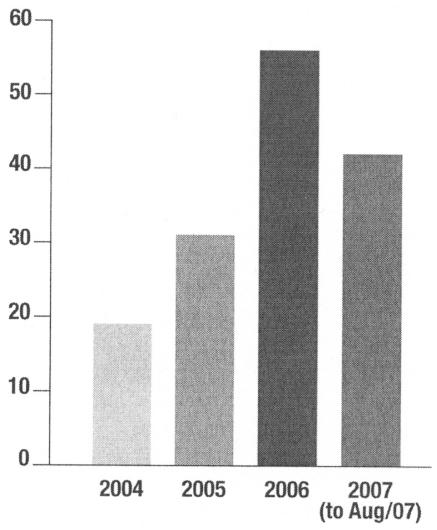
*Number of multicentre cancer protocols submitted annually. Adapted, with permission, from* Ontario Cancer Research Ethics Board: 2006 Annual Report 2.

In 2004, five sites used ocreb as their reb of record, and ocreb received 19 multicentre cancer protocols 2. At the end of 2005, the number of institutions using ocreb had increased to seven, and the number of new submissions, to 31. By the end of 2006, the number of institutions authorizing a formal reb-of-record relationship with ocreb had doubled to fourteen, and 56 new submissions had been received. Today, ocreb is being used solely as a reb of record, and by August 2007, it had reviewed another 42 multicentre cancer protocols (Figure 1). Table II lists the centres that have authorized their institutions to use ocreb as reb of record on a study-by-study basis.

## 3. PROOF OF CONCEPT

The proof of ocreb’s success lies not only in the increase in submissions to the board, but also in the testimonies of users and in their assessments of ocreb’s ability to deal with the challenges faced by the burgeoning organization.

Dr. Amit Oza, one of the three pis with the pmh Phase ii Consortium group, attests to the fact that ocreb has achieved the difficult task of securing the trust of local institutional rebs and of pis. Princess Margaret Hospital has the distinction of being the first centre to use ocreb, initially for facilitated review but later as reb of record for pmh Phase ii Consortium trials, and the Consortium is therefore well positioned to assess ocreb’s progress. “Local institutional review boards take the responsibility to their institution very seriously, so handing off that responsibility to ocreb was a very big leap of faith,” says Oza.

**FIGURE 2:**
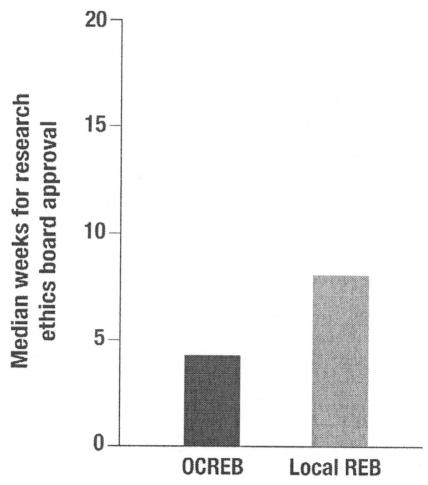
Time to research ethics board (reb) approval.

Although ocreb was rapidly adopted by nonacademic hospitals with limited expertise in cancerspecific research ethics, larger academic hospitals were slower to follow suit. Some even made the significant investment of testing the waters by establishing parallel submission for a time to assess how ocreb would fare. In doing so, they learned that ocreb had the capacity to shave three weeks off trial startup time (Figure 2).

In Oza’s opinion, ocreb “met the challenge of building up its resources over time, and is now more efficient than any other reb.” He views ocreb’s greatest strengths as “streamlining the administrative process through multiple sites, getting trials opened at sites simultaneously, and the quality of its reviews.”

Connie Dupuis, country study manager at Hoffmann–La Roche, is also an advocate of ocreb. She sees the organization as having “demonstrated its commitment to a centralized research ethics review board function in Ontario. The single review on behalf of several centres has the potential to be more efficient than several individual submissions and reviews in terms of time and resources, and has the potential to shorten study start-up timelines and support clinical trials completion.”

Academic investigators and industry sponsors alike are encouraged that ocreb has developed a workable solution to the challenge of reporting saes. Serious and unexpected aes are reported by the sponsors to regulatory agencies such as Health Canada and to every researcher testing the same drug in any study. These reports—termed “external saes”—flood in from around the world. Before submitting these external sae reports to their reb, trial investigators must photocopy the multiple pages of each report and then create, complete, and sign a summary form indicating that they have reviewed the documents.

Dozens of sae reports per study may arrive every month, with the investigator often receiving the same report for multiple studies that include the same investigational agent. The investigator and the reb can both soon become afloat in a sea of saes. Although saes are universally recognized as occupying a place of central importance in protecting patient safety, the cumbersome process of reporting them is considered by all concerned to be the bane of clinical trials.

The simple, yet clever, approach designed by ocreb to balance patient safety with the redundancy of sae reporting is to

- move toward electronic submission of saes (previously reported by hard copy).
- request that the sponsor submit the collated saes directly to ocreb with the acknowledgment that the site investigators must continue to receive and review them.
- provide investigators with access to a summary of the reported saes for each investigational agent.

Hoffmann–La Roche agreed to ocreb’s request and is now using an automated electronic system to send collated saes simultaneously to ocreb and site investigators. Dupuis says that the company is “very pleased to do this, because it substantially reduces time and resources for both the centres and our clinical monitors.” Oza sees the potential for ocreb to reduce the burden at individual sites and to effectively manage the volume of saes.

## 4. SUMMARY

The critical role played by rebs cannot be overstated: without them, clinical trials would grind to a halt. Currently, 1335 oncology clinical trials are underway in Canada, 430 of them in Ontario 3,4. Of the 430, some 80% are multicentre cancer trials. The bright future for ocreb within the vast landscape of such trials rests in having the people, policies, and procedures in place to streamline the submission and ongoing review processes, while maintaining the highest level of human participant protection. The ability of ocreb to shorten and coordinate study startup times imparts a competitive edge to Ontario, not only making the province an attractive location in which to conduct cancer clinical trials, but also improving access to novel therapies for patients with limited treatment alternatives.

In Ontario, ocreb is poised for the substantial growth required to oversee the ever increasing number of cancer clinical trials. Throughout Canada, ocreb is being embraced as a viable model for provincial rebs, primarily because it is viewed as a “central-reb model that works.”

## Figures and Tables

**TABLE I:** Centres collaborating to form the Ontario Cancer Research Ethics Board

Cancer Care Ontario
National Council for Ethics in Human Research
Canadian Association of Research Ethics Boards
Canadian Institutes of Health Research

**TABLE II:** Institutions using the Ontario Cancer Research Ethics Board

Trillium Health Centre (Mississauga)
Thunder Bay Regional Health Sciences Centre Cancer Care Program
Southlake Regional Health Centre (Newmarket)
Windsor Regional Cancer Centre
Toronto East General Hospital
Princess Margaret Hospital (Toronto)
Juravinski Cancer Centre (Hamilton)
Grand River Regional Cancer Centre (Kitchener)
Cambridge Memorial Hospital
Odette Cancer Centre a (Toronto)
Mount Sinai Hospital (Toronto)
Ottawa Hospital Regional Cancer Centre
Niagara Health Sciences (St. Catherines)
London Regional Cancer Program

a Formerly Sunnybrook Regional Cancer Centre.

## Footnotes

a Saginur R, Dent S, Schwartz L, Heslegrave R, Stacey S, Manzo J. The Ontario Cancer Research Ethics Board (ocreb): a multicentre regional irb. In preparation.
